# The distinct roles of PTHrP and PTH1R in long bone growth and digit formation

**DOI:** 10.1093/jbmrpl/ziag119

**Published:** 2026-07-26

**Authors:** Takeshi Shimoide, Loan Nguyen-Yamamoto, Lucie Canaff, Naoyuki Kawao, Yuto Takada, Hiroshi Kaji, Akifumi Enomoto, David Goltzman

**Affiliations:** Department of Oral and Maxillofacial Surgery, Kindai University Faculty of Medicine, Osaka 590-0197, Japan; Research Institute of the McGill University Health Centre and Department of Medicine, McGill University, Montreal, Quebec H4A 3J1, Canada; Research Institute of the McGill University Health Centre and Department of Medicine, McGill University, Montreal, Quebec H4A 3J1, Canada; Department of Physiology and Regenerative Medicine, Kindai University Faculty of Medicine, Osaka 590-0197, Japan; Department of Oral and Maxillofacial Surgery, Kindai University Faculty of Medicine, Osaka 590-0197, Japan; Department of Physiology and Regenerative Medicine, Kindai University Faculty of Medicine, Osaka 590-0197, Japan; Department of Oral and Maxillofacial Surgery, Kindai University Faculty of Medicine, Osaka 590-0197, Japan; Research Institute of the McGill University Health Centre and Department of Medicine, McGill University, Montreal, Quebec H4A 3J1, Canada

**Keywords:** parathyroid hormone-related protein, receptor, parathyroid hormone, type 1, mesenchymal progenitor cells, skeletal development, digit patterning, morphogenesis

## Abstract

PTH-related peptide (PTHrP) and its receptor, the PTH/PTHrP receptor type 1 (PTH1R), are essential for chondrocyte differentiation and epiphyseal cartilage homeostasis. To evaluate the functional differences between PTHrP and PTH1R derived from mesenchymal progenitor cells (MPCs) in skeletal development, we generated MPC-specific *Pthrp* conditional KO mice (PTHrP cKO; *Prrx1-Cre*; *Pthrp*^flox/flox^) or *Pth1r* cKO mice (PTH1R cKO; *Prrx1-Cre*; *Pth1r*^flox/flox^) using the Prrx1-Cre driver and analyzed their skeletal phenotypes. Both PTHrP cKO mice and PTH1R cKO mice exhibited severe shortening of long bones and impaired growth plate formation immediately after birth. However, in the digits, only PTHrP cKO mice showed distinctive digit deviations in the toes, which were similar to those seen in human diastrophic dysplasia (DTD). These results suggest that MPC-derived PTHrP and PTH1R play distinct roles. Both are essential for longitudinal bone growth, but PTHrP plays an important role in regulating toe position and morphology. This phenotypic difference suggests a novel PTH1R-independent function of PTHrP in skeletal development. Thus, our study provides genetic evidence for this mechanism, offering insights into the pathogenesis of congenital digit deformities such as those seen in DTD.

## Graphical Abstract

**Graphical Abstract:**
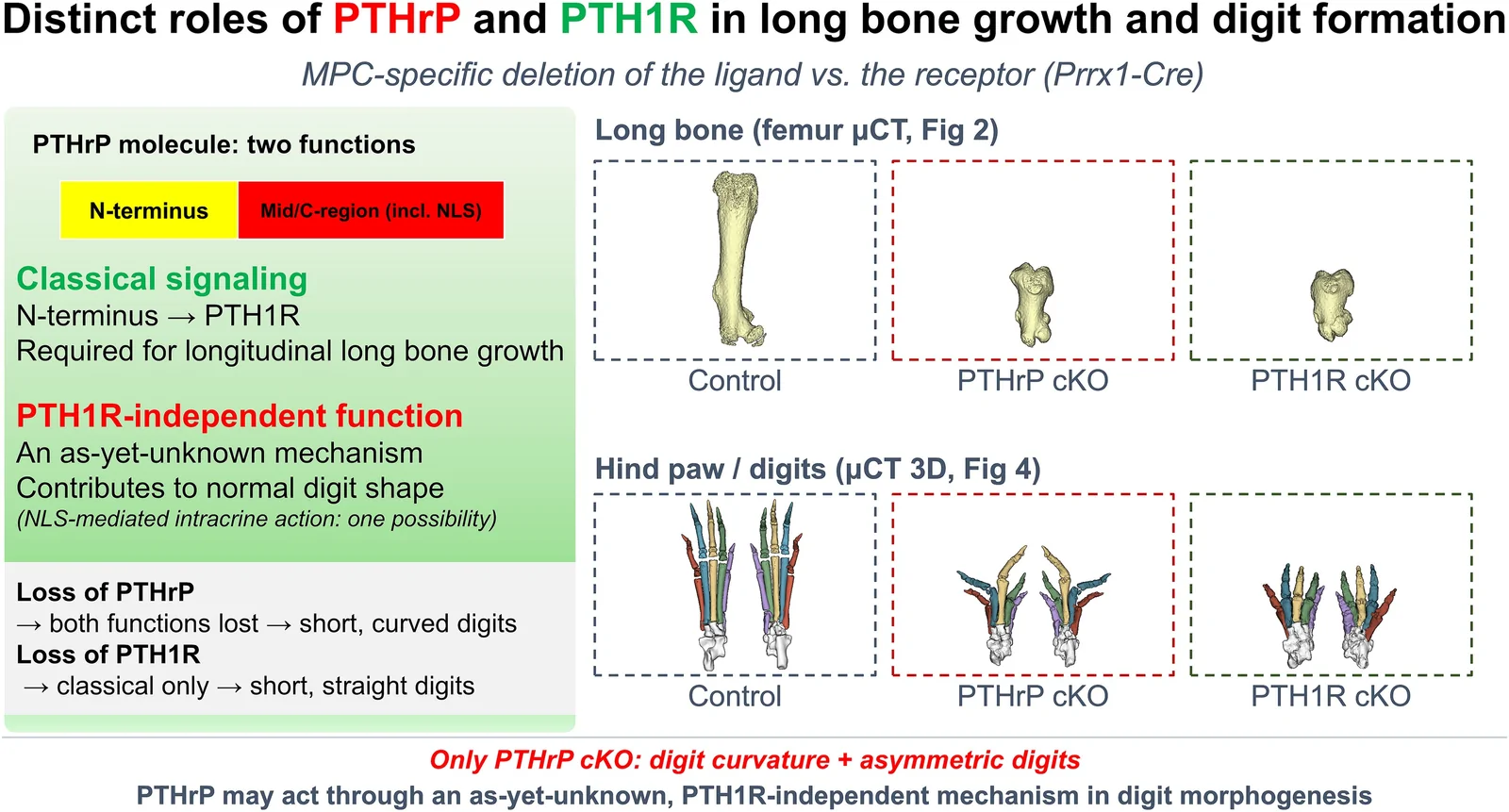

## Introduction

The development and growth of long bones are achieved through a series of processes involving the proliferation, differentiation, and hypertrophy of chondrocytes in the growth plate cartilage.^1^^,^^2^ These processes are strictly regulated in both space and time. In this regulatory mechanism, the signaling pathway composed of PTH-related peptide (PTHrP), acting via its NH_2_-terminal domain, and its primary G protein-coupled receptor, the PTH/PTHrP receptor type 1 (PTH1R), is recognized as one of the most important regulatory mechanisms.^3^ During development, PTHrP is mainly secreted from quiescent and proliferative layer chondrocytes in the perichondrium and growth plate and acts in a paracrine mode on neighboring chondrocytes via PTH1R. This signaling inhibits the differentiation of chondrocytes to the hypertrophic phase and promotes proliferation.^4^ Furthermore, the expression of PTHrP is positively regulated by Indian hedgehog, and the negative feedback loop formed by these two molecules helps maintain the structural and functional homeostasis of the growth plate.^5^^,^^6^ Mice with systemic deficiency of *Pthrp* or *Pth1r* show severe chondrodysplasia and die perinatally due to respiratory failure related to skeletal dysplasia. However, the physiological role of PTHrP cannot be fully explained by classical extracellular signal transduction via PTH1R alone. Indeed, PTHrP has been reported to slow vascular invasion of the early cartilage model by a mechanism independent of PTH1R.^7^ Furthermore, the mid-region of the PTHrP molecule contains a functional nuclear localization sequence (NLS), and PTHrP may directly translocate into the nucleus after being internalized into cells, where it may exert an “intracrine” function by regulating nuclear events such as apoptosis and cell proliferation.^8–10^ The physiological significance of this PTH1R-independent “non-classical function” is strongly supported by studies using knock-in mice expressing PTHrP that retain only the N-terminal region and lack the middle-to-C-terminal region containing the NLS. Despite producing N-terminal PTHrP, which activates PTH1R, the mice display lethal chondrodysplasia similar to that observed in global PTHrP KO mice.^11^^,^^12^ This observation suggests that domains of PTHrP beyond the N-terminus exert functions independent of classical signaling pathways involving PTH1R, but that are required for skeletal development.

Thus, it is essential to understand which cells the non-N-terminal region of PTHrP interacts with. Mesenchymal progenitor cells (MPCs) are common precursor cells for chondrocytes and osteoblasts and play a central role in maintaining skeletal homeostasis from limb development through postnatal skeletal growth and repair/regeneration.^13^ The specific roles played by PTHrP in MPCs through both classical and non-classical pathways remain unclear. Several clues point to the importance of PTHrP/PTH1R signaling in the MPC lineage. In studies using a Prrx1-Cre driver specific to MPCs, Pth1r-deficient mice were generated in MPCs and exhibited skeletal abnormalities such as shortening of long bones and symphalangism.^14^^,^^15^ These studies therefore suggested the importance of the PTH1R signaling pathway in MPCs and contributed to elucidating the physiological role of the PTHrP/PTH1R signaling pathway in this cell lineage. However, the role of the PTHrP molecule itself, produced by MPCs, has not been extensively analyzed. Thus, no studies exist that inhibit either the entire function or the receptor function of PTHrP within the same MPC cell lineage and compare their phenotypes. Such comparative analyses are necessary to distinguish between the PTH1R-mediated function of PTHrP and potential non-classical functions inherent to the PTHrP molecule itself. The role of PTHrP in processes such as long bone elongation has been addressed; however, the unique contribution of PTHrP to the complicated morphogenesis responsible for digit bone shape has not been evaluated. This study was designed to test the central hypothesis that PTHrP possesses novel PTH1R-independent functions in complex skeletal development. To this end, we generated conditional KO (cKO) mice for MPC-specific *Pthrp* or *Pth1r* and performed detailed comparative analyses of their postnatal skeletal phenotypes, focusing on long bone growth and toe morphogenesis.

## Materials and methods

### Animals and husbandry

All animal experiments were conducted in accordance with the guidelines of, and were approved by, the IACUCs of McGill University Health Centre and Kindai University. *Pthrp*^flox/flox,^^16^  *Pth1r*^flox/flox,^^17^ and *Prrx1-Cre* mouse lines have been previously described.^18^  *Prrx1-Cre* mice (Stock No: 005584) were obtained from The Jackson Laboratory (Bar Harbor). *Pthrp*^flox/flox^ mice were a kind gift from Dr. Andrew C. Karaplis (McGill University), and *Pth1r*^flox/flox^ mice were a kind gift from Dr. Henry M. Kronenberg (Massachusetts General Hospital). To generate MPC-specific cKO mice, Prrx1-Cre transgenic mice were crossed with mice carrying floxed alleles of *Pthrp* and *Pth1r*. The resulting *Prrx1-Cre*; *Pthrp*^flox/+^ males were then crossed with *Pthrp*^flox/flox^ females to obtain PTHrP cKO (*Prrx1-Cre*; *Pthrp*^flox/flox^) mice. Using the same breeding method, we generated PTH1R cKO (*Prrx1-Cre*; *Pth1r*^flox/flox^) mice. Cre-negative littermates (*Pthrp*^flox/flox^ or *Pth1r*^flox/flox^) served as controls. Because of the heterozygous crossing scheme, each litter contained both normal-sized Cre-negative pups and much smaller cKO pups, and the small pups were at a disadvantage during nursing. To help the small pups survive, we time-mated several dams of the same genotype so that litters were born at the same time, and then redistributed the pups by body size at 1-2 d of age. The smaller pups (presumed cKO) were nursed by one dam and the larger Cre-negative pups by another, which made the litter sizes more even and reduced competition among pups for milk. Foster dams were of the same mixed background as the colony. Animals were housed in specific pathogen-free facilities under a 12-h light-dark cycle with ad libitum food and water. Mice of the same genotype were maintained and analyzed at the same age (3 wk) at both institutions; X-ray and length measurements were performed on samples from McGill University, and micro-CT analysis on samples from Kindai University. Offspring were reared under their parents or foster mothers and were fed as needed. Both male and female pups were analyzed. No significant sex differences were observed in skeletal morphology at 3 wk of age, so data from both sexes were combined. Following the 3Rs principle, the minimum number of animals necessary to achieve statistical significance was used. Group sizes varied, and the exact n is given in each figure legend. The limb deformities were consistent among all cKO mice. Thus, these numbers were sufficient to characterize the phenotype.

### Genotyping

Genomic DNA was extracted from tail biopsies, and routine PCR was used to genotype the various mice. The following primer sequences were used to perform PCR analyses: *Pthrp*-forward, 5′-GAGGCTAAGCCAGGAGGATT-3′, *Pthrp*-reverse, 5′-CCCCATCCTCTCTCCTCTCT-3′; *Pth1r*-forward, 5′-ATGAGGTCTGAGGTACATGGCTCTGA-3′, *Pth1r*-reverse, 5′-CCTGCTGACCTCTCTGAAAGAATGT-3′; and *Prrx1-Cre*-mutant-forward, 5′-GCGGTCGGCAGTAAAAACTATC-3′ (oIMR1084), *Prrx1-Cre*-mutant-reverse, 5′-GTGAAACAGCATTGCTGTCACTT-3′ (oIMR1085). Internal control primers for *Prrx1-Cre* genotyping were 5′-CTAGGCCACAGAATTGAAAGATCT-3′ (oIMR7338) and 5′-GTAGGTGGAAATTCTAGCATCATCC-3′ (oIMR7339). PCR conditions included 30 cycles with an annealing temperature of 56 °C for floxed alleles, or followed the Jackson Laboratory protocol (Stock No. 005584) for the Prrx1-Cre Tg. Product sizes were identified as ~150 bp (WT) and ~279 bp (floxed) for *Pthrp*, ~300 bp (WT) and ~400 bp (floxed) for *Pth1r*, and ~100 bp (mutant) and ~324 bp (control) for Prrx1-Cre.

### Skeletal image analysis

Hindlimbs from 3-wk-old mice were dissected, and X-ray analysis was performed at the McGill University Centre for Advanced Bone and Periodontal Research using the Kubtec Xpert 80 system (22 kV, 540 μA; Kubtec). Femur length was measured using ImageJ/Fiji (NIH). BMD and BMC were measured by dual-energy X-ray absorptiometry (DXA) using the PIXImus II densitometer (GE Lunar).

### Micro-CT and image analysis

Dissected femurs and hindlimbs from 3-wk-old mice were scanned with a CosmoScan GX II (Rigaku) operating at 90 kV and 88 μA, with a 10-mm field of view, a 20-μm isotropic voxel size, and the high-resolution scan mode (4-min scan; X-ray filter, Cu 0.06 mm + Al 0.5 mm; ring-artifact reduction applied). All samples were scanned under identical conditions, and 3D surface reconstructions were performed using a 3D Slicer (v. 5.10). For quantitative analysis using BoneJ (Fiji/ImageJ), cortical and trabecular bones were segmented applying global gray-value thresholds of 850 and 700, respectively, within a display range of −1300 to 3000.

Due to the marked difference in femoral length between control (~10 mm) and cKO (~4 mm) mice, ROI sizes were adjusted proportionally as recommended by Bouxsein et al. (2010). Using the distal articular surface as a landmark, the trabecular ROI was uniformly set from 15% to 25% of the total femoral length. This starting position corresponded to 5 slices (0.1 mm) distal to the growth plate in control mice. Trabecular parameters [BV/TV, trabecular thickness, trabecular separation (Tb.Sp), and trabecular number (Tb.N)] were evaluated using the Thickness and Volume Fraction modules. This range corresponded to about 50 slices (~1.0 mm) in control femurs and about 20 slices (~0.4 mm) in cKO femurs, representing the same relative 10% of bone length in each group. Cortical parameters [cortical thickness (Ct.Th), cortical bone area, cross-sectional area (Tt.Ar), and cortical area fraction (Ct.Ar/Tt.Ar)] were measured at the mid-diaphysis (50% of the femoral length) with the Slice Geometry module. All measurements were performed by an investigator who was blinded to the genotypes of the mice.

### Histological analysis

The PLP solution fixed hind limbs at 4 °C for three days to prepare them for histological examination. The PLP solution required 100 mL of 8% paraformaldehyde (Sigma, P-6148), 300 mL of 0.1 M L-lysine (Sigma, L-5501), and 856 mg of sodium periodate (Sigma, S-1878) to create the fixative, which was adjusted to pH 7.4. The samples underwent decalcification in an EDTA-glycerol solution (29 g EDTA, 3 g NaOH, 30 mL glycerol in 200 mL water; pH 7.3) at 4 °C for 2 wk after their fixation period. The solution needed replacement every two to three days during the decalcification process. Samples underwent washing followed by ethanol dehydration steps before paraffin embedding. For paraffin histology, the decalcified samples were embedded in paraffin and cut into 4-μm longitudinal sections, which were stained with H&E or Alcian blue/Kernechtrot. For von Kossa/toluidine blue staining, bones were fixed in 70% ethanol, embedded in MMA without decalcification, and sectioned.

### Statistical analysis

All data are represented as means ± SD. The Tukey’s *post hoc* test following one-way ANOVA determined significant differences between groups. All statistical analyses were performed using Prism 8 statistical software (GraphPad Software, Inc.). Differences among experimental groups were considered significant when the *p*-value was <.05. The number of animals used for each analysis is indicated in the figure legends.

## Results

### Mesenchymal progenitor cell-derived PTHrP and PTH1R are essential for long bone growth and growth plate formation

**Figure 1:**
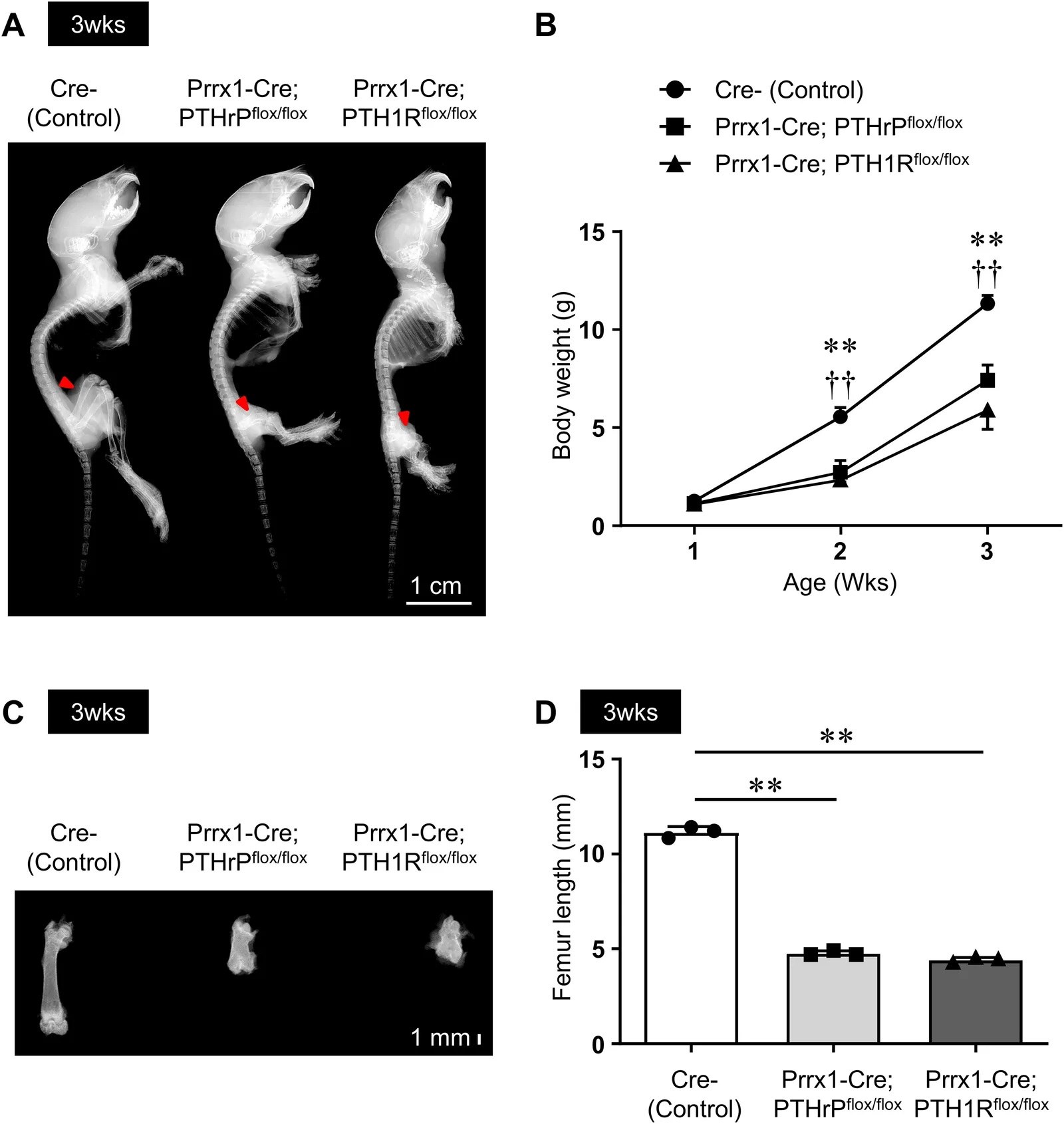
MPC-specific deletion of *Pthrp* or *Pth1r* results in severe postnatal growth retardation in mice. (A) Whole-body plain radiographs of 3-wk-old control (Cre−), PTHrP cKO (*Prrx1-Cre*; *Pthrp*^flox/flox^), and PTH1R cKO (*Prrx1-Cre*; *Pth1r*^flox/flox^) mice. (B) Growth curves showing BW of control, PTHrP cKO, and PTH1R cKO mice at 1, 2, and 3 wk of age. (C) Plain radiographs of femurs from 3-wk-old mice. (D) Quantification of femur length. Data are presented as mean ± SD (*n* = 3 per group). Symbols denote the genotype in (B): ● control (Cre–), ■ PTHrP cKO, ▲ PTH1R cKO. Statistical comparisons are vs control: ^*^p < .05 and ^**^p < .01 for control vs PTHrP cKO; † *p* < .05 and †† *p* < .01 for control vs PTH1R cKO, by one-way ANOVA followed by Tukey’s multiple comparison test.

To elucidate the roles of PTHrP and PTH1R in MPCs for skeletal development, we generated MPC-specific *Pthrp* KO (PTHrP cKO: *Prrx1*-Cre; *Pthrp*^flox/flox^) and *Pth1r* KO (PTH1R cKO: *Prrx1*-Cre; *Pth1r*^flox/flox^) mice. Heterozygous cKO mice (*Prrx1*-Cre; *Pthrp*^flox/+^, *Prrx1*-Cre; *Pth1r*^flox/+^) showed a similar phenotype to their Cre-negative littermates, and no evidence of haploinsufficiency was observed. We first analyzed the effects on long bone skeletal growth. Severe limb shortening was a common phenotype in both PTHrP cKO and PTH1R cKO mice at 3 wk of age, compared to control (Cre−) littermates (Figure 1A). Both cKO groups displayed similar growth retardation, reducing BW and femur length to ~60% and ~45% of controls, respectively, with no significant difference between the two groups (Figure 1B-D).

**Figure 2:**
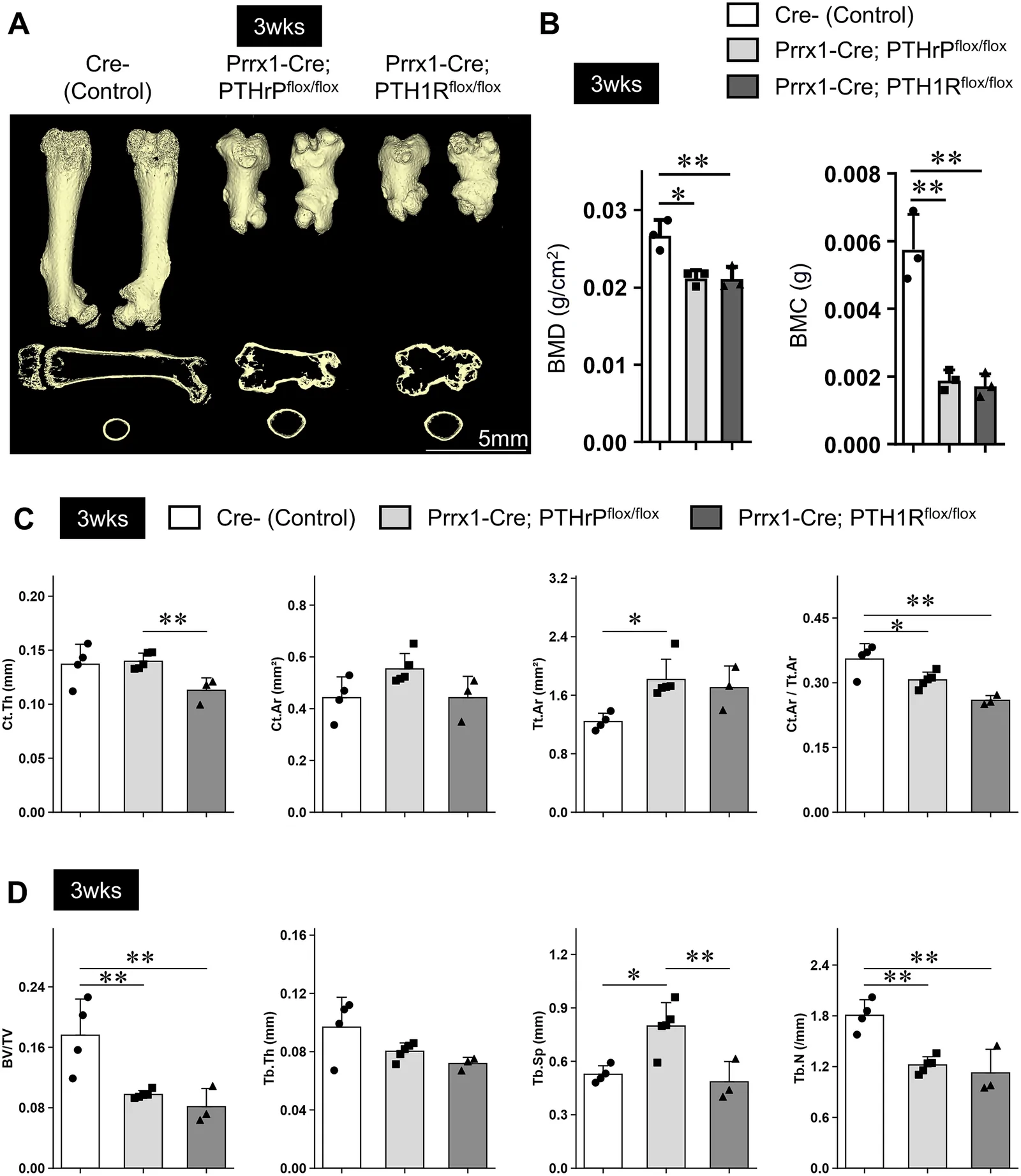
Micro-CT analysis reveals impaired long bone formation in both PTHrP cKO and PTH1R cKO mice. (A) Representative micro-CT images of the femur from 3-wk-old control (Cre−), PTHrP cKO, and PTH1R cKO mice. The top row shows three-dimensional surface reconstructions; the middle and bottom rows show single longitudinal and transverse sections through the mid-diaphysis, respectively. All images are shown at the same magnification; scale bar, 5 mm. (B) BMD and BMC of the femur, measured by DXA. (C) Cortical bone parameters measured at the mid-diaphysis: cortical thickness (Ct.Th), cortical bone area (Ct.Ar), total cross-sectional area (Tt.Ar), and cortical area fraction (Ct.Ar/Tt.Ar). (D) Trabecular bone parameters measured in the distal femur (15%-25% of the total femoral length from the distal articular surface): bone volume fraction (BV/TV), trabecular thickness (Tb.Th), trabecular separation (Tb.Sp), and trabecular number (Tb.N). Data are presented as mean ± SD (*n* = 4 [Control], 5 [PTHrP cKO], 3 [PTH1R cKO]). ^*^p < .05, ^**^p < .01 (one-way ANOVA with Tukey’s *post hoc* test).

Micro-CT (μCT) and histological analyses revealed the cause of this growth disorder. Three-dimensional reconstruction and sectional μCT images of the femur showed marked growth plate dysplasia in both cKO groups (Figure 2A). Dual-energy X-ray absorptiometry analysis showed that BMD and BMC were significantly reduced in both cKO groups (Figure 2B). Quantitative μCT analysis supported these findings. In the cortical bone, the Ct.Ar/Tt.Ar was reduced in both cKO groups, while the total Tt.Ar tended to be larger than in controls (Figure 2C). Thus, the cKO femurs were short and wide, as if the metaphyseal width was retained without diaphyseal elongation. In the trabecular bone, bone volume fraction (BV/TV) and Tb.N were decreased in both cKO groups (Figure 2D). Some parameters, such as Ct.Th and Tb.Sp, differed between the two cKO groups, but the overall μCT profiles were largely comparable, and no clear difference in longitudinal growth was observed.

**Figure 3:**
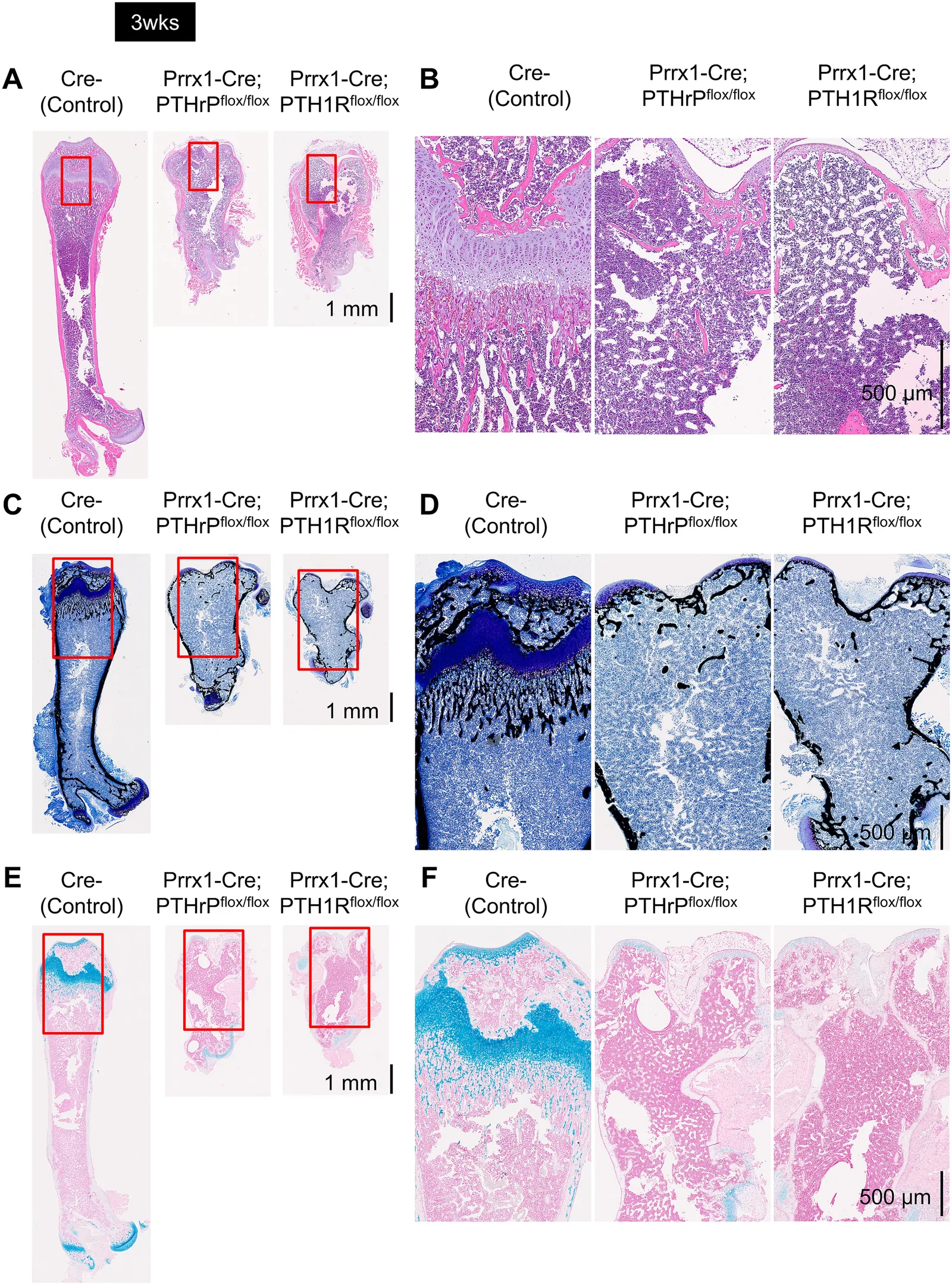
Histological analyses reveal severely impaired growth plate formation but preserved articular cartilage in the femurs of both PTHrP cKO and PTH1R cKO mice. Longitudinal sections of the distal femur from 3-wk-old control (Cre−), PTHrP cKO (*Prrx1*-Cre; *Pthrp*^flox/flox^), and PTH1R cKO (*Prrx1*-Cre; *Pth1r*^flox/flox^) mice (*n* = 3 per genotype). Representative images are shown. (A) H&E staining, low-magnification overview. Boxed regions indicate the distal growth plate region enlarged in (B). (B) High-magnification H&E images of the boxed region in (A). Both cKO groups lack the organized columnar arrangement of growth plate chondrocytes seen in controls. (C) von Kossa/toluidine blue staining, low-magnification overview. Boxed regions indicate the region enlarged in (D). (D) High-magnification von Kossa/toluidine blue images of the boxed region in (C), showing disorganization of the growth plate cartilage and loss of the normal columnar structure in both cKO groups. The articular cartilage at the distal femur is morphologically preserved in both cKO groups, comparable to controls. (E) Alcian blue/Kernechtrot staining, low-magnification overview. Boxed regions indicate the region enlarged in (F). (F) High-magnification Alcian blue/Kernechtrot images of the boxed region in (E), demonstrating reduced and disorganized cartilage matrix in the growth plate of both cKO groups. All images are from 3-wk-old mice. Scale bars: 1 mm (A, C, E) and 500 μm (B, D, F).

Consistent with these findings, H&E staining showed that both cKO groups lacked the columnar chondrocyte structure seen in controls, indicating severely impaired growth plate formation (Figure 3A and B). von Kossa/toluidine blue and Alcian blue/Kernechtrot staining also showed disorganized growth plate cartilage and loss of the normal columnar arrangement in both cKO groups (Figure 3C-F). These results indicate that PTHrP/PTH1R signaling is essential for both the longitudinal growth of long bones and the maintenance of the growth plate. In contrast, the articular cartilage at the distal femur was preserved in both cKO groups, comparable to controls (Figure 3D).

### Specific PTH1R-independent PTHrP function in toe morphogenesis

**Figure 4:**
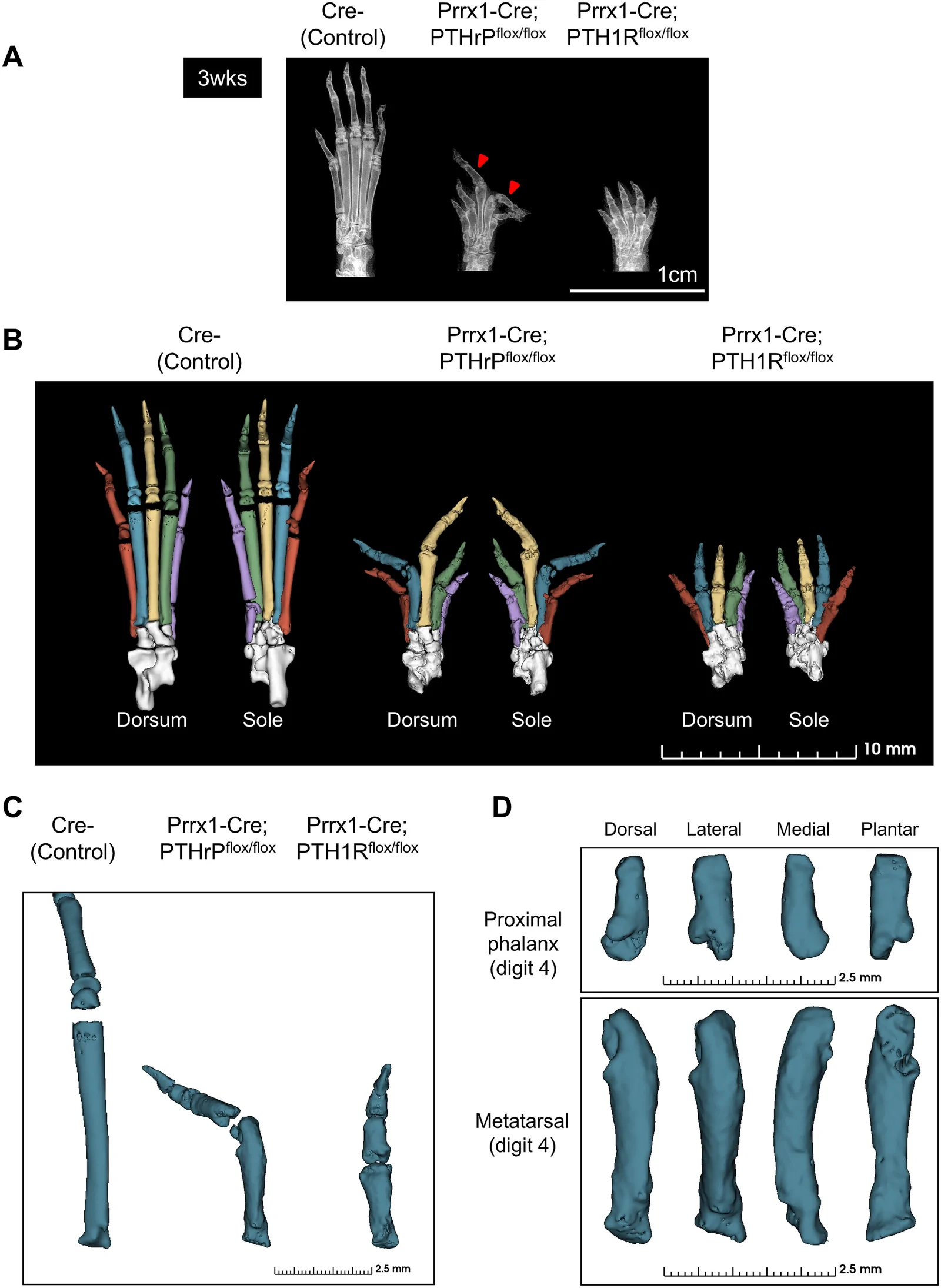
PTHrP cKO mice, but not PTH1R cKO mice, develop a distinct digit curvature in the hindlimb. Skeletal analysis of the right hind paw from 3-wk-old control (Cre−), PTHrP cKO (*Prrx1*-Cre; *Pthrp*^flox/flox^), and PTH1R cKO (*Prrx1*-Cre; *Pth1r*^flox/flox^) mice. (A) Plain radiographs of the right hind paw. Arrowheads indicate the digit curvature seen only in the PTHrP cKO mouse. Scale bar, 1 cm. (B) Three-dimensional micro-CT surface reconstructions of the right hind paw, shown in dorsal and plantar sole views. Each digit is pseudo-colored to help identify the individual rays. The digits of the PTHrP cKO mouse were shortened and deviated, with a widened gap between the third and fourth digits, whereas those of the PTH1R cKO mouse were shortened but remained straight. Scale bar, 10 mm. (C) Isolated micro-CT reconstructions of the metatarsal and proximal phalanx of the fourth digit in each genotype, centered on the joint between them. In the PTHrP cKO, these bones were angulated at this joint, producing the curvature, whereas those of control and PTH1R cKO mice remained straight. Scale bar, 2.5 mm. (D) The fourth-digit bones (metatarsal and proximal phalanx) of a PTHrP cKO mouse were isolated and rotated about the long axis to examine their three-dimensional shape. Each bone is shown in four orientations: dorsal, lateral, medial, and plantar. Upper row, proximal phalanx; lower row, metatarsal. Both bones showed a left-right (medio-lateral) asymmetry of the bone surface, which may underlie the curvature of the digit. Scale bars, 2.5 mm. Representative images are shown (*n* = 3 per genotype). Lengths of the metatarsals and phalanges for all digits are quantified in Table 1.

In contrast to their shared requirement in the long bones, loss of the ligand and loss of the receptor produced strikingly different phenotypes in the digits of the hind paw. Both cKO genotypes showed phalangeal shortening, but only PTHrP cKO mice developed a deformity characterized by pronounced curvature and flexion of the digits (Figure 4A). All PTHrP cKO mice consistently developed a widened gap between the third and fourth digits. At birth the digits were indistinguishable from those of PTH1R cKO mice; the curvature emerged at approximately 1 wk of age and was fully established by 3 wk. The site and direction of the curvature were highly consistent across all PTHrP cKO mice examined from different litters (*n* = 15). This deformity was completely absent in control mice and in PTH1R cKO mice, even though PTH1R cKO mice exhibited brachydactyly of comparable severity.

Three-dimensional micro-CT reconstruction of the whole hind foot showed that the digits of PTHrP cKO mice were both shortened and angularly deviated, whereas those of PTH1R cKO mice were shortened but remained straight (Figure 4B). Measurement of the hind paw digits showed that the digits were shortened in both cKO genotypes (Table 1). For the fourth digit, the metatarsal and proximal phalanx of control mice measured 6.85 ± 0.35 and 3.00 ± 0.21 mm, and were reduced in PTHrP cKO mice to 3.37 ± 0.34 and 1.52 ± 0.07 mm, and in PTH1R cKO mice to 2.11 ± 0.05 and 1.13 ± 0.06 mm, respectively (ANOVA *p* < .001). Although both genotypes were markedly shortened overall, in the third and fourth digits—where the curvature occurred—the bones of PTHrP cKO mice were not shorter than, and for the third metatarsal were clearly longer than, those of PTH1R cKO mice (Figure 4B, Table 1). Thus, shortening alone did not account for the curvature unique to PTHrP cKO mice.

**Table 1:** Lengths of the hind paw metatarsals and phalanges in control, *PTHrP* cKO, and *PTH1R* cKO mice. For each digit of the hind paw, the lengths of the metatarsal and of the phalanges were measured at 3 wk of age and are shown as mean ± SD (mm). Digit 1 has only two phalanges (proximal and distal) and no middle phalanx, so no middle phalanx is listed for digit 1.

**Digit**	**Bone**	**Control**	**PTHrP cKO**	**PTH1R cKO**	**ANOVA *p***	***Post hoc***
		(mm)	(mm)	(mm)		
**Digit 1 (hallux)**	Distal phalanx	0.93 ± 0.15	0.70 ± 0.05	0.57 ± 0.11	.020	ns / ^*^ / ns
	Proximal phalanx	1.79 ± 0.33	0.76 ± 0.06	0.63 ± 0.13	<.001	^**^ / ^**^ / ns
	Metatarsal	3.83 ± 0.38	1.06 ± 0.17	1.11 ± 0.11	<.001	^**^ / ^**^ / ns
**Digit 2**	Distal phalanx	1.01 ± 0.13	0.80 ± 0.15	0.59 ± 0.16	.036	ns / ^*^ / ns
	Middle phalanx	0.85 ± 0.22	0.68 ± 0.21	0.42 ± 0.04	.063	ns / ns / ns
	Proximal phalanx	2.91 ± 0.07	1.04 ± 0.09	0.99 ± 0.10	<.001	^**^ / ^**^ / ns
	Metatarsal	6.73 ± 0.24	2.05 ± 0.12	1.76 ± 0.03	<.001	^**^ / ^**^ / ns
**Digit 3**	Distal phalanx	1.02 ± 0.29	0.81 ± 0.22	0.62 ± 0.21	.212	ns / ns / ns
	Middle phalanx	0.98 ± 0.22	0.72 ± 0.07	0.45 ± 0.06	.010	ns / ^**^ / ns
	Proximal phalanx	3.00 ± 0.13	1.61 ± 0.04	1.08 ± 0.09	<.001	^**^ / ^**^ / ^**^
	Metatarsal	6.80 ± 0.50	4.14 ± 0.02	1.88 ± 0.04	<.001	^**^ / ^**^ / ^**^
**Digit 4**	Distal phalanx	0.96 ± 0.19	0.77 ± 0.16	0.60 ± 0.13	.084	ns / ns / ns
	Middle phalanx	1.01 ± 0.20	0.76 ± 0.10	0.58 ± 0.09	.026	ns / ^*^ / ns
	Proximal phalanx	3.00 ± 0.21	1.52 ± 0.07	1.13 ± 0.06	<.001	^**^ / ^**^ / ^*^
	Metatarsal	6.85 ± 0.35	3.37 ± 0.34	2.11 ± 0.05	<.001	^**^ / ^**^ / ^**^
**Digit 5**	Distal phalanx	0.79 ± 0.22	0.57 ± 0.12	0.46 ± 0.07	.080	ns / ns / ns
	Middle phalanx	0.84 ± 0.25	0.57 ± 0.03	0.55 ± 0.04	.088	ns / ns / ns
	Proximal phalanx	2.36 ± 0.05	0.99 ± 0.16	1.00 ± 0.12	<.001	^**^ / ^**^ / ns
	Metatarsal	5.38 ± 0.47	1.73 ± 0.05	2.20 ± 0.03	<.001	^**^ / ^**^ / ns

Data are mean ± SD; *n* = 3 mice per group. Lengths are given in millimeters (mm).

Lengths were measured on micro-CT three-dimensional reconstructions using ImageJ/Fiji (NIH) after calibration to the scale bar.

Differences among the three groups were assessed for each bone by one-way ANOVA followed by Tukey’s *post hoc* test, using Prism 8 (GraphPad Software).

The *post hoc* column shows the three pairwise comparisons in the order control vs *PTHrP* cKO/control vs *PTH1R* cKO/*PTHrP* cKO vs *PTH1R* cKO.

^*^ *p* < .05; ^**^p < .01; ns, not significant.

To investigate the structural basis of this curvature, we isolated the metatarsal and proximal phalanx of the fourth digit and reconstructed each separately (Figure 4C). In control and PTH1R cKO mice, these bones were straight and symmetric and differed only in length. In PTHrP cKO mice, however, the bones were angulated at the joint, with misalignment of the adjoining articular surfaces, reproducing the curvature observed in the intact paw. When the isolated bones were rotated and viewed from the dorsal, lateral, medial, and plantar aspects, those of PTHrP cKO mice showed a consistent left-right (medio-lateral) asymmetry of the bone surface, which was not observed in the other genotypes (Figure 4D).

**Figure 5:**
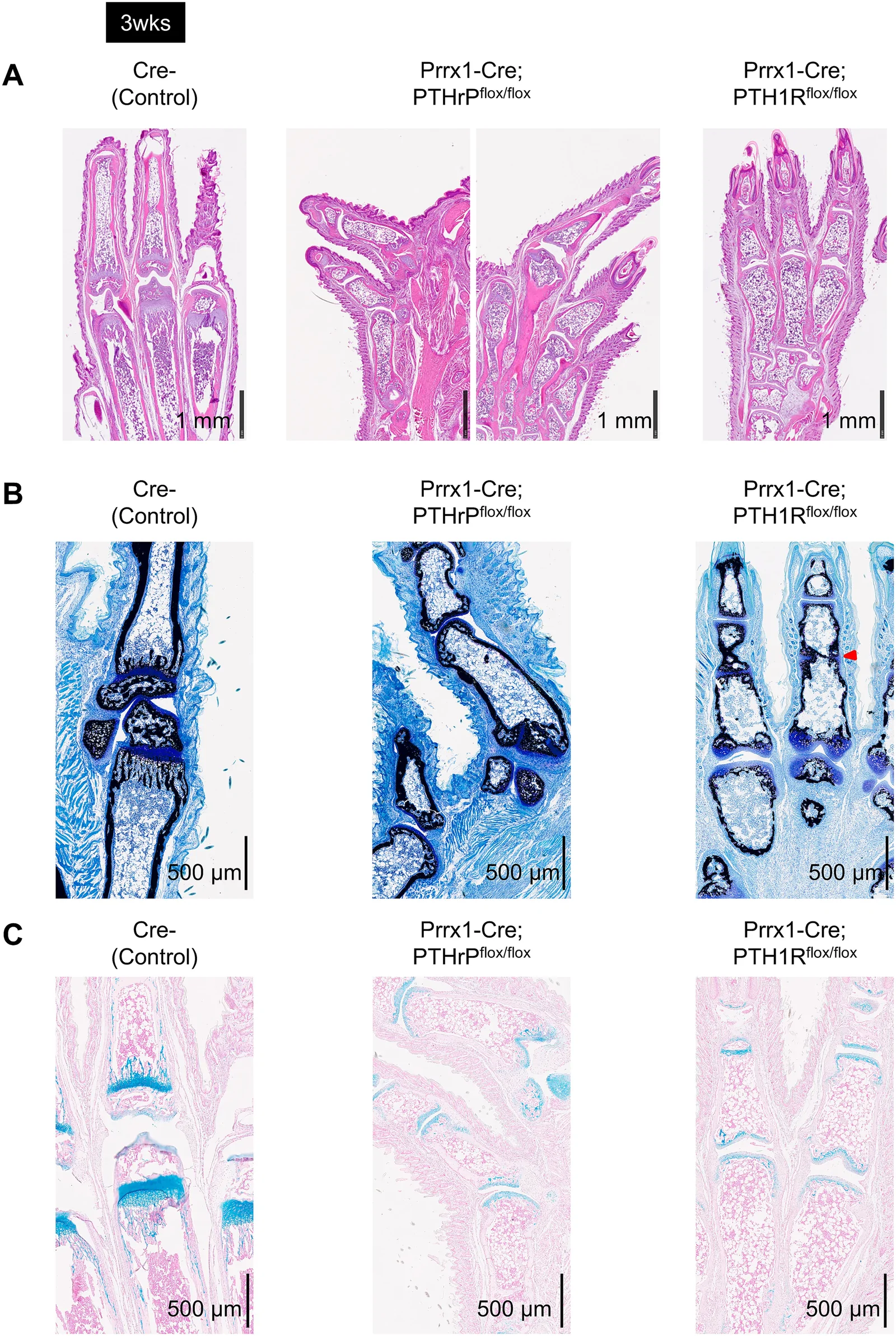
Histological analysis of growth plate and articular cartilage in cKO digits. Longitudinal sections of the digits (hind paw) from 3-wk-old control (Cre−), PTHrP cKO (*Prrx1*-Cre; *Pthrp*^flox/flox^), and PTH1R cKO (*Prrx1*-Cre; *Pth1r*^flox/flox^) mice (n = 3 per genotype). Representative images are shown. (A) H&E staining. The foot bones of control and PTH1R cKO mice were straight, whereas those of PTHrP cKO mice were deformed with misaligned joints. Articular cartilage was present in all groups. (3D reconstructed images are shown in Figure 4.) (B) von Kossa/toluidine blue staining. Both cKO models showed disorganized mineralization and disrupted growth plates, but the articular cartilage looked normal. The arrowhead indicates phalangeal fusion (symphalangism) in the PTH1R cKO digit. (C) Alcian blue/Kernechtrot staining. The cartilage matrix (glycosaminoglycans) of the growth plate was markedly reduced in both cKO groups compared with controls, whereas the articular cartilage staining was preserved. All images are from 3-wk-old mice. Scale bars: 1 mm (A) and 500 μm (B, C).

This asymmetric bone shape likely represents at least one structural basis for the digit curvature, and histology supported this. H&E staining showed that the digits of PTHrP cKO mice were angulated, with misaligned joints and wedge-shaped epiphyses, whereas those of control and PTH1R cKO mice remained straight; articular cartilage was present at the joint surfaces in all genotypes (Figure 5A). In both cKO genotypes, the digits showed disorganized mineralization, loss of the normal columnar growth-plate structure, and a marked reduction in growth-plate cartilage matrix, while the articular cartilage was relatively preserved (Figure 5B and C). In the PTH1R cKO digits, adjacent phalanges appeared fused, consistent with the symphalangism reported by Amano *et al*.^14^ (Figure 5B, arrowhead). The interphalangeal joint space in PTHrP cKO digits also appeared slightly wider, suggesting that peri-articular soft tissues, such as the joint capsule, ligaments, and articular cartilage, may contribute to the deformity together with the asymmetric bone shape. In the forelimbs, the humerus, radius, and ulna were also markedly shortened in both cKO groups, and the forelimb digits were shortened as well; unlike the hind paw, however, they did not show the curvature seen in PTHrP cKO mice.

## Discussion

The fact that bone shortening occurred similarly in both cKO mouse groups and no difference in length was observed suggests that PTHrP and PTH1R are equally essential for long bone elongation and that PTHrP/PTH1R signaling is involved in the longitudinal development of bone. The micro-CT data support this idea. In both cKO groups, the Ct.Ar/Tt.Ar was reduced and the total Tt.Ar tended to be larger, while the bone volume fraction (BV/TV) and Tb.N was decreased. These femurs were short and wide, and the two genotypes were similar. Even though the growth plate was severely disrupted, the articular cartilage at the distal femur was preserved in both groups. This suggests that PTHrP and PTH1R are mainly needed for the growth plate, not for the articular cartilage. Conversely, the fact that the ligand and receptor KO phenotypes diverge so clearly in toe tissues strongly suggests that PTHrP possesses an unknown function independent of PTH1R-mediated signaling in the process of establishing normal digit morphology.

To interpret these two distinct phenomena, it is necessary to consider whether the phenotypic difference between the two cKO mice could be due to technical issues in Cre recombinase efficiency. Prrx1-Cre activity is known to be mosaic-like in the hindlimbs compared to the forelimbs,^18^ and it has also been reported that recombination efficiency varies between loci.^20^ If toe curvature were attributable to mosaic Cre activity or reduced Cre activity efficiency at the Pth1r locus, the toe phenotype in PTHrP cKO mice would likely appear as a milder defect than that seen in Pth1r cKO mice, and the phenotype would be expected to be present from early embryonic stages. However, contrary to this expectation, PTHrP cKO mice exhibited a characteristic “deformation” rather than simple toe shortening. Furthermore, this deformation progressed particularly rapidly in the early postnatal period (1-3 wk), and the location and direction of toe curvature were consistent across more than 10 mice (including those from different breeders). Such high consistency and postnatal onset suggest this is not a technical artifact. Instead, it reflects a biological function of PTHrP, independent of PTH1R, in regulating toe morphology. When we measured the hind paw digits, both cKO groups showed marked shortening of the metatarsals and phalanges. However, in the third and fourth digits, where the curvature occurred, the bones of PTHrP cKO mice were no shorter than—and, for the third metatarsal, longer than—those of PTH1R cKO mice (Table 1). The curvature therefore cannot be attributed to a greater degree of shortening; on the contrary, it developed even though these digits were relatively longer in PTHrP cKO mice. To look at this more closely, we isolated the metatarsal and proximal phalanx of the fourth digit and reconstructed them in three dimensions. In PTHrP cKO mice, each bone was angled at the joint and showed a left-right asymmetry of the bone surface, whereas the same bones in control and PTH1R cKO mice were straight and symmetric (Figure 4C and D). This asymmetric bone shape is likely one structural basis of the curvature. Alcian blue/Kernechtrot staining showed that the growth-plate cartilage matrix in the digits was reduced and disorganized in both cKO groups, while the articular cartilage was relatively preserved (Figure 5C). The interphalangeal joint space in PTHrP cKO digits also looked slightly wider, so soft tissues around the joint may also contribute to the deformity together with the asymmetric bone shape.

**Figure 6:**
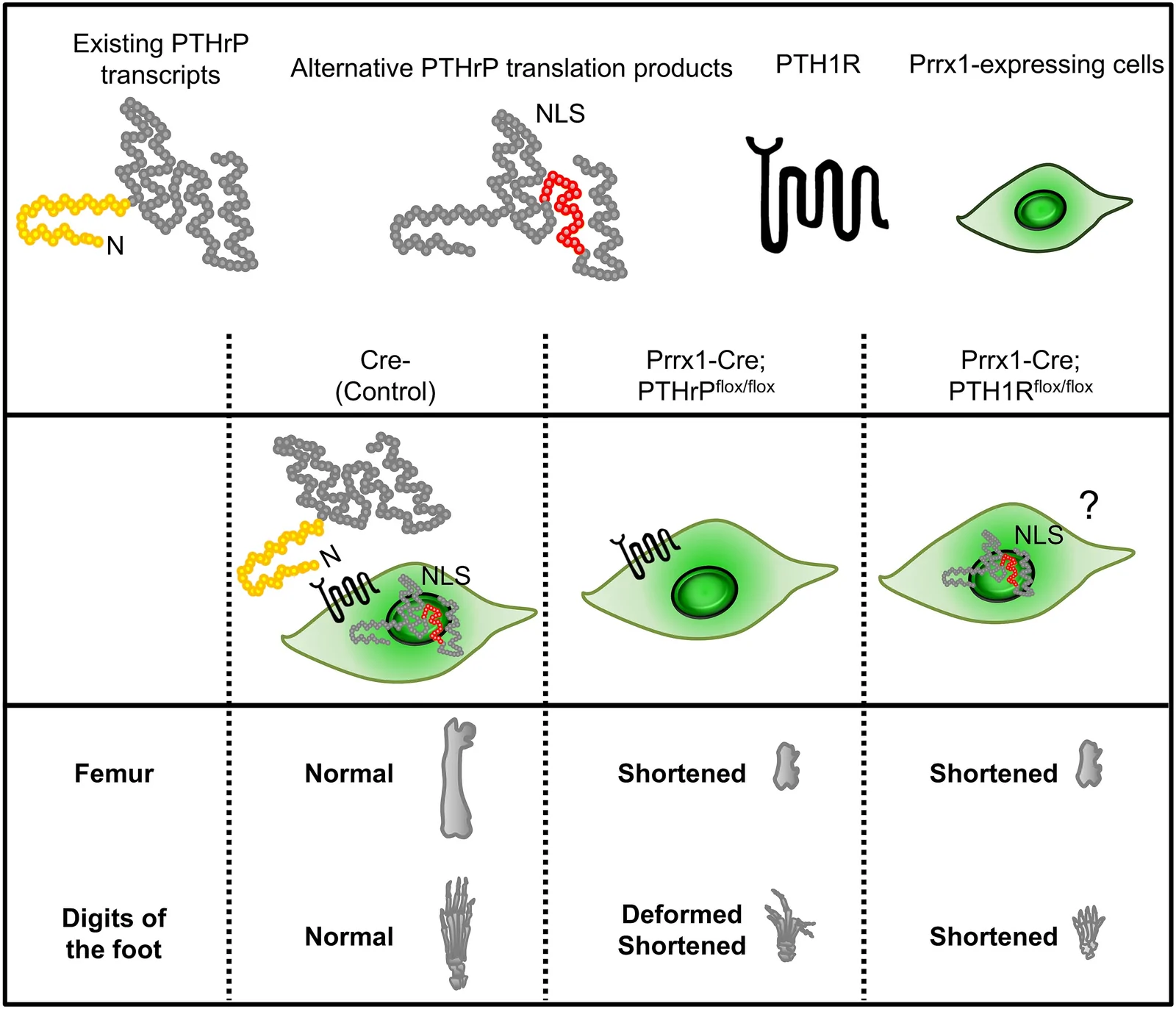
Dual-function model of MPC-derived PTHrP in bone formation. This study suggests that classical signaling via the N-terminus of PTHrP with PTH1R is essential for longitudinal growth of long bones. However, since PTHrP could not bind PTH1R, which was deleted, and since PTHrP cannot bind PTH2R, it is likely that the NLS domain, which can exhibit *in vivo* functionality,^12^ is needed for normal phalangeal morphogenesis. Loss of PTHrP inhibits both functions, causing phalangeal shortening and deformity. In contrast, loss of PTH1R abolishes only the classical function of PTHrP, leading to digit shortening without phalangeal curvature. The N-terminal domain of PTHrP is denoted in yellow. NLS (nuclear localization sequence) is denoted in red.

Interestingly, this phalangeal curvature was not observed in the forelimbs. While purely speculative at this stage, the distinct transcriptional environments created by Tbx5 (forelimb) and Tbx4 (hindlimb) could potentially account for this hindlimb-specific requirement.^21^^,^^22^ Therefore, the PTH1R-independent function of PTHrP might be specific to the Tbx4-regulated hindlimb environment. However, we cannot rule out other possibilities. Soft tissues, such as tendons, muscles, and nerves, may also contribute to the deformity. Future studies should determine whether PTHrP targets chondrocytes or surrounding tissues.

The molecular entity responsible for this PTH1R-independent function is most likely the NLS present in the central region of the PTHrP molecule, which mediates nuclear function.^9^^,^^10^^,^^12^^,^^19^ PTHrP that has translocated into the nucleus may directly regulate the expression of specific gene clusters involved in the three-dimensional pattern formation of digits by interacting with transcription factors and chromatin modification factors. This hypothesis is consistent with previous studies using knock-in mice, which have already suggested the importance of the region containing the NLS of PTHrP.^10^^,^^12^^,^^19^ Alternatively, the possibility that PTHrP binds to unknown cell surface receptors or interacts with other intracellular proteins cannot be completely ruled out. It is also an important finding that toe deformities become prominent not at birth but during subsequent growth. This suggests that the PTH1R-independent function of PTHrP is not required for early embryonic patterning. Instead, it becomes critical after birth to maintain cartilage structure and ensure the toes grow in the correct direction. Based on these observations, hypotheses regarding the underlying mechanisms can be formulated. For example, postnatal curvature may result from an imbalance in cellular activities, such as asymmetric proliferation and apoptosis of chondrocytes on either side of the phalanges, or from remodeling of the extracellular matrix, or both. Elucidating these events represents the next critical step. Based on this, our prediction that intralaminar interactions of the NLS domain provide the molecular basis for this role offers a crucial framework for investigating how PTHrP regulates these postnatal processes. Future studies, including the generation of targeted NLS mutant mice, will be essential to directly test this specific mechanism. Nevertheless, the distinct postnatal phenotype we have revealed provides important rationale for these future mechanistic studies. Finally, this PTHrP cKO mouse-specific phenotype is similar to the finger deformities “Hitchhiker’s Thumb” observed in human skeletal dysplasia caused by mutations in the sulfate transporter gene (SLC26A2), known as diastrophic dysplasia.^23–25^ Since abnormal sulfation of proteoglycans is known to lead to cartilage formation defects, it is possible that PTHrP is also involved in maintaining the homeostasis of the cartilage extracellular matrix through this non-canonical pathway. Although the underlying mechanisms remain to be determined, this study provides definitive genetic evidence for a novel function of PTHrP independent of PTH1R. This study clearly demonstrates the phenotypic separation between ligands and receptors, highlights the multifaceted biological functions of PTHrP, demonstrates a previously unknown function of PTHrP, and offers a novel viewpoint for elucidating the diverse roles played by a single molecule in complex organ development, thereby providing a mechanistic framework to understand skeletal anomalies that do not map to the canonical receptor dysfunctions (Figure 6).

## Supplementary Material

ARRIVE_Animals_in_Research_Reporting_In_Vivo_Experiments_ziag119

## Data Availability

The data that support the findings of this study are available from the corresponding author upon reasonable request.
