# Supplementary material for: The distinct roles of PTHrP and PTH1R in long bone growth and digit formation
Source: JBMR Plus. 2026 Jul 26;10(9):ziag119. doi: 10.1093/jbmrpl/ziag119 (PMC13496395; doi:10.1093/jbmrpl/ziag119)
Supplement: Supplementary file 1 [file arrive_animals_in_research_reporting_in_vivo_experiments_ziag119.pdf]

2001 K Street, NW,
3rd Floor North
Washington, DC
20006, USA

P: 202-367-2336
E: jbmr@asbmr.org

Corresponding Author Name: Takeshi Shimoide

Manuscript Title: The distinct roles of PTHrP and PTH1R in long bone growth and digit fc

Animal Research: Reporting of *In Vivo* Experiments (ARRIVE) Guideline Requirements

Completion of the ARRIVE checklist helps achieve reproducibility for manuscripts reporting results of experiments involving animals by listing the kinds of information needed to ensure that such manuscripts contain the rationale and limitations underlying the experimental and statistical approach, and the information needed to reproduce the reported experiments and comparison with previous studies. The checklist thus aids authors in providing detailed information within their submission that meets a standard to achieve reproducibility and transparency of research, and assists reviewers in their effort determine whether the necessary information is present. If you have any questions, please contact us at jbmr@asbmr.org.

JBMR® has adapted the ARRIVE guidelines. To fulfill the guideline requirements for JBMR®, please select the statement that describes your study and fill out only the required ARRIVE checklist questions for the study.

☐ ARRIVE CHECKLIST A- In my study, animals are used only as a source of tissues, cells, or other materials for subsequent *in vitro* experiments and no *in vivo* data are reported.

☑ ARRIVE CHECKLIST B- In my study, animals *in vivo* data are reported.

ARRIVE CHECKLIST A

Animals are used only as a source of tissues, cells, or other materials for subsequent *in vitro* experiments and no *in vivo* data are reported.

Recommendation	Page
Title/Abstract- State the species of animal used	3
Introduction- Explain how the animals being used will meet the needs of the scientific objectives; and when appropriate, state the study's relevance to human biology.	6
Methods-<br>Indicate the nature of the ethical review permissions, relevant licenses and national or institutional guidelines for the care and use of animals that cover the research.<br><br>Provide details of the animals used, including source of animals, the species, strain, substrain, sex, and age or developmental stage, genetic modification status, and health/ immune status.	8
Methods- Provide details of other relevant information on housing, husbandry conditions, and diet.	8

ARRIVE CHECKLIST B

Animal *in vivo* data are reported.

Recommendation	Page
Title/Abstract- Provide details of the species or strain of animal used	3
Introduction- Explain how the animals used will meet the needs of the scientific objectives. If appropriate, state the study's relevance to human biology.	6
Methods- Indicate the nature of the ethical review permissions, relevant licenses and national or institutional guidelines for the care and use of animals that cover the research.	8
For each experiment and treatment group, provide precise details of all animal procedures.<br>State how animals were allocated to treatment groups. If a specific allocation procedure was used, describe it.<br>State whether investigators were blinded during allocation, animal handling, and endpoint measurements.<br>Identify the source of administered substances.<br>* For prospective preclinical studies, describe the statistical approach used to determine sample size and power (a post-experiment calculation of power is not acceptable), and define the primary and secondary experimental outcomes.	8
Provide details of the animals used, including source of animals, the species, strain, sub-strain, sex, age or developmental stage, genetic modification status, and health/immune status.	8
Provide details of housing (gang or single), husbandry conditions (e.g. specific pathogen free), and diet including commercial source and product number.	8
Report the exact number (not range) of animals in each group for each experimental outcome.<br>For prospective preclinical studies, report relevant characteristics and health status of animals (e.g. weight, microbiological status, if relevant, and drug or test naïve, previous procedures) prior to treatment or testing.<br>Specify the unit of analysis for each dataset (e.g. single animal, group of animals). Provide details of the statistical methods used for each analysis. Describe methods used to assess whether the data met the assumptions of the statistical approach.	8
Give details of all important adverse events in each experimental group. Describe any modifications to the experimental protocols made to reduce adverse events.	8
Discussion- Comment on any limitations of the animal model.	13
